# Graphene–graphite hybrid epoxy composites with controllable workability for thermal management

**DOI:** 10.3762/bjnano.10.9

**Published:** 2019-01-08

**Authors:** Idan Levy, Eyal Merary Wormser, Maxim Varenik, Matat Buzaglo, Roey Nadiv, Oren Regev

**Affiliations:** 1 Department of Chemical Engineering, Ben-Gurion University of the Negev, Beer-Sheva 84105, Israel; 2 Department of Chemistry, Nuclear Research Center Negev, P.O.B.9001, Beer-Sheva 84190, Israel; 3 Department of Materials and Interfaces, Weizmann Institute of Science, Rehovot 7610001, Israel,; 4 Ilse Katz Institute for Nanoscale Science and Technology, Ben-Gurion University of the Negev, Beer-Sheva 84105, Israel

**Keywords:** hybrid composites, nanocomposites, rheology, thermal interface material, thermal properties

## Abstract

The substantial heat generation in highly dense electronic devices requires the use of materials tailored to facilitate efficient thermal management. The design of such materials may be based on the loading of thermally conductive fillers into the polymer matrix applied – as a thermal interface material – on the interface between two surfaces to reduce contact resistance. On the one hand, these additives enhance the thermal conductivity of the composite, but on the other hand, they increase the viscosity of the composite and hence impair its workability. This in turn could negatively affect the device–matrix interface. To address this problem, we suggest a tunable composite material comprising a combination of two different carbon-based fillers, graphene nanoplatelets (GNPs) and graphite. By adjusting the GNP:graphite concentration ratio and the total concentration of the fillers, we were able to fine tune the thermal conductivity and the workability of the hybrid polymer composite. To facilitate the optimal design of materials for thermal management, we constructed a ‘concentration–thermal conductivity–viscosity phase diagram’. This hybrid approach thus offers solutions for thermal management applications, providing both finely tuned composite thermal properties and workability. We demonstrate the utility of this approach by fabricating a thermal interface material with tunable workability and testing it in a model electronic device.

## Introduction

Modern-day miniaturization of electronic devices [1] goes hand in hand with the demand for increased performance, which, in turn, leads to high-power consumption and consequently to substantial heat generation. Thermal management of miniaturized electronic devices thus poses a significant challenge [2]. To efficiently dissipate heat to the environment, the potting material used in the device must be thermally conductive from the heat source to a cooling device, be it a passive heat sink utilizing a large surface area, or an active cooling system [1]. Critical requirements for the potting material are thus high bulk thermal conductivity and minimal contact resistance between the heat source and the cooling device.

Directly adjoining the coarse surfaces of the heat source and the cooling device will generally result in poor contact and entrapment of thermally insulating air. To address this problem, a thermal interface material (TIM) [3] is applied at the interface between the two surfaces to reduce the contact resistance. Commonly used types of TIM [4–6] include thermal greases and pastes, solder, phase-change materials [7] and, very often, filled-polymer adhesives, which are usually epoxy-based [5,8–12].

Suitable fillers for such polymer-based TIM composites, whether metallic, ceramic or graphitic, should exhibit excellent thermal conductivity (TC), exceeding 100 W/(m∙K). In recent years, boron nitride (BN) (TC = 360 W/(m∙K) [13]) has been employed as a filler in polymer-based composites, displaying high TC enhancement, although at high loading [14–15].

Some graphitic fillers have theoretical TC values of up to several thousands of W/(m∙K) [16–17], making them natural candidates for use in TIMs. Within the group of graphitic fillers, it seemed likely that carbon nanotubes (CNTs) would be suitable materials by virtue of their high TC (>3500 W/(m∙K) for individual tubes) [18–19], but their performance has proved to be disappointing [20–23] as a result of phonon scattering at the tube–tube interface. Another graphitic candidate that appears to have good potential as a filler material is graphene, a two-dimensional sheet of sp^2^-hybridized carbons, with a much lower filler-to-filler resistance than that of the CNTs [11,24–25]. In recent years, extensive studies have been conducted on graphite and graphene nanoplatelets (GNPs, composed of several graphene layers, with thickness of up to ≈100 nm) [26] as fillers [10,12,27–37]; it has been shown that these materials enhance the TC of polymer-based TIMs by almost two orders of magnitude [25]. These results are superior over other carbon allotropes such as nanodiamonds [38–39].

With that being said, solder TIM may reach thermal conductivities at least one order-of-magnitude higher than those of carbon-based composites [6]. However, modern trends for minimization and the production of ultra-lightweight electronic devices reject the use of high-density metallic composites, preferring lower-density carbon. Therefore, a carbon-based composite is an attractive alternative for the production of miniature electronic devices with specific thermal properties.

An important consideration in the design of filler materials is the possibility that the TC of the TIM could be degraded over time, particularly as a result of the cycling between high and low working temperatures that is typical in electronic devices [2]. In solid TIMs, delamination, which can occur due to differences in thermal expansion between the substrate and the TIM, will introduce thermally insulating air voids into the interface. In liquid or paste-like TIMs, differences in thermal expansion between the hot and cold surfaces could result in the TIM leaking out of the interface, thus increasing the contact resistance. These malfunctions in the performance of TIMs could stem from their high viscosity values during application on surfaces [40]. It is, therefore, important to tailor the rheological properties of the TIM to the specific application, while still striving to maintain a high TC in the bulk material.

In a previous study [41] we have demonstrated that by loading a polymer matrix with two fillers, namely, GNP and graphite, it is possible to produce composite with highly tunable rheological properties for thermal management applications [42–45]. In this work, we focused on developing a highly applicable composite material by enhancing the thermal properties of an epoxy polymer that is commonly used as a matrix for TIM applications, while maintaining desirable rheological properties [5,9–12,46]. In this study, a broader picture was obtained on the integration of graphite–GNP fillers and on the impact of viscosity on optimal design of composite materials for thermal management applications.

The effect of the composite viscosity on the TC of the applied TIM was investigated both via electron microscopy and by fabrication of a proof-of-concept setup for functional setting, demonstrating that the rheological properties of the TIM can have an effect on the contact resistance, and thus on the overall thermal conductivity in real-life TIM applications. In light of our findings, we are now in a position to provide a ‘road map’ for designing a hybrid composite in which both viscosity and TC may be tuned for thermal management applications.

## Results and Discussion

We explore how the workability of a filled matrix can be controlled by using a combination of two fillers, namely, graphite and GNP, and compare the workability and TC values to those obtained for a single-filler and hybrid composites.

We start by characterizing the filler dispersion quality in the polymer matrix. Then, the thermal conductivity and rheological properties of single-filler composites will be characterized. This will be followed by a study of hybrid composites, highlighting the implications of the rheological properties on thermal management applications.

### Filler characterization within the epoxy matrices

**Figure 1:**
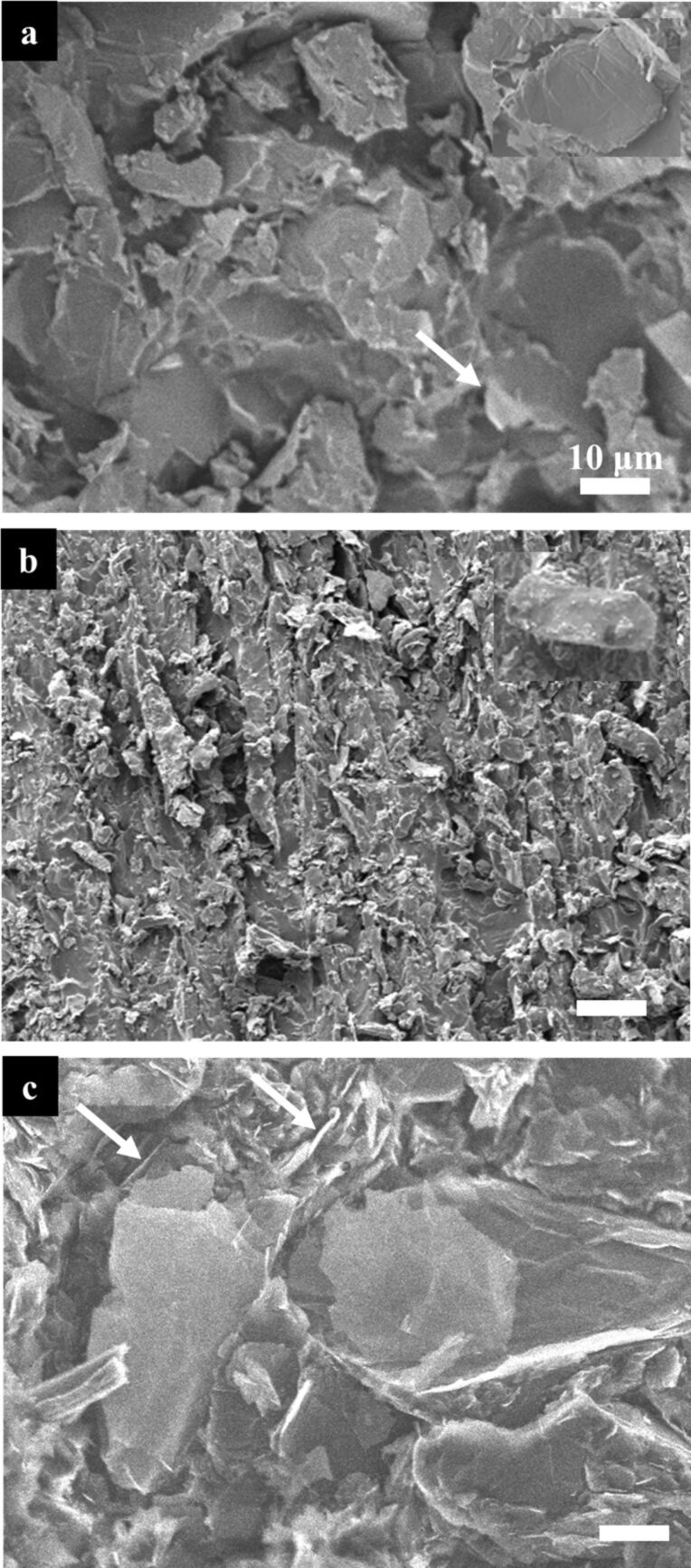
SEM images of graphene nanoplatelet- (a) and graphite- (b) loaded single-filler epoxy composites. (c) GNP–graphite hybrid composite. The arrows indicate edge-on GNP filler in (a) and (c).

The average length of the filler particles was determined by measuring 100 particles for each filler material (Section S1 in Supplementary Information File 1). The sizes of the different fillers, when imbedded in the epoxy matrix, are 19 ± 3 µm and 27 ± 4 µm for the GNP and the graphite, respectively (Figure 1a,b and Supplementary Information File 1, Figure S2a,b). The GNPs in the composite are mostly edge-on (arrows in Figure 1a and 1c), where the graphite is thicker and rather bulky. Homogenous filler-dispersion patterns are revealed in the epoxy matrices of each of the three systems (Figure 1a–c).

### Thermal conductivity and rheology of single-filler composites

**Figure 2:**
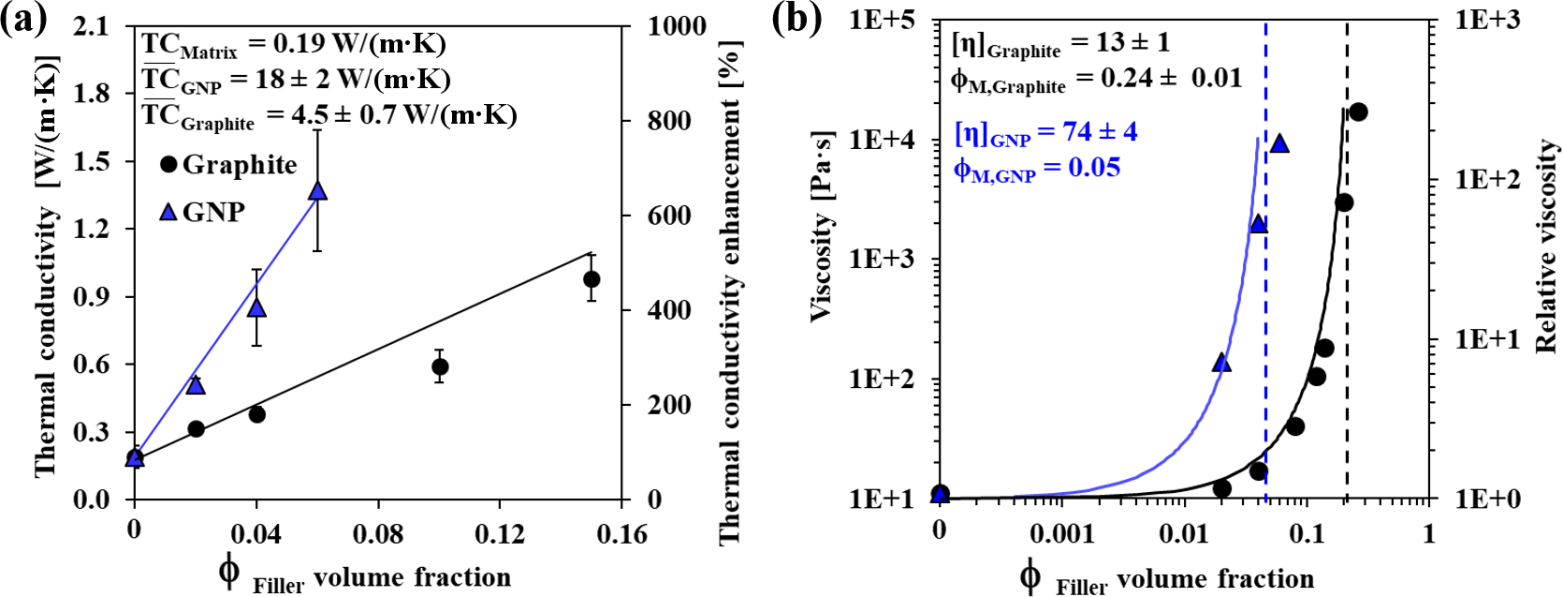
Single-filler composites: (a) TC and (b) viscosity and relative viscosity as functions of filler type and volume fraction at constant shear rate (0.1 1/s) and temperature of 25 °C. Each graph displays the fitting parameters. The solid lines in (a) are fits to the ROM model (Equation 1), and those in (b), to the Krieger–Dougherty model (Equation 2). The dashed lines in (b) represent the critical volume fraction, φ_M_, found by fitting to the Krieger–Dougherty (K–D) model (Equation 2). Some error bars are hidden by the data symbols due to small measurement errors.

For composites loaded with a single filler (either GNP or graphite), the TC shows a linear dependence with the volume fraction of the filler (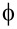
_Filler_, Figure 2a). An enhancement of about ≈700% and ≈500% in the TC of the single-filler composite vs the neat epoxy was obtained when the matrix was loaded with GNPs or graphite at volume fractions of 0.05 and 0.15, respectively (Figure 2a).

The TC of a composite, TC_Composite_, may be represented by the rule of mixtures (ROM; Equation 1):

[1] $${\text{TC}}_{\text{Composite}}={\text{TC}}_{\text{Matrix}}\cdot \phi_{\text{Matrix}}+\sum_{\text{Filler}}{\bar{\text{TC}}}_{\text{Filler}}\cdot \phi_{\text{Filler}}$$

The TC of the composite, TC_Composite_, is calculated from the arithmetic mean of the TC of the filler-free matrix, TC_Matrix_ [20] and the effective TC of the filler, 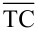
_Filler_. The value of 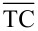
_Filler_ is much lower than the theoretical value (see Figure 2a and Table 1 in the Experimental section) for both GNPs and graphite due to filler–matrix and filler–filler contact resistances [46]. Since both TC_Composite_ and TC_Matrix_ are measured quantities (see Experimental section), the value of 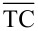
_Filler_ can be extracted for graphite and for GNPs (Figure 2a).

**Figure 3:**
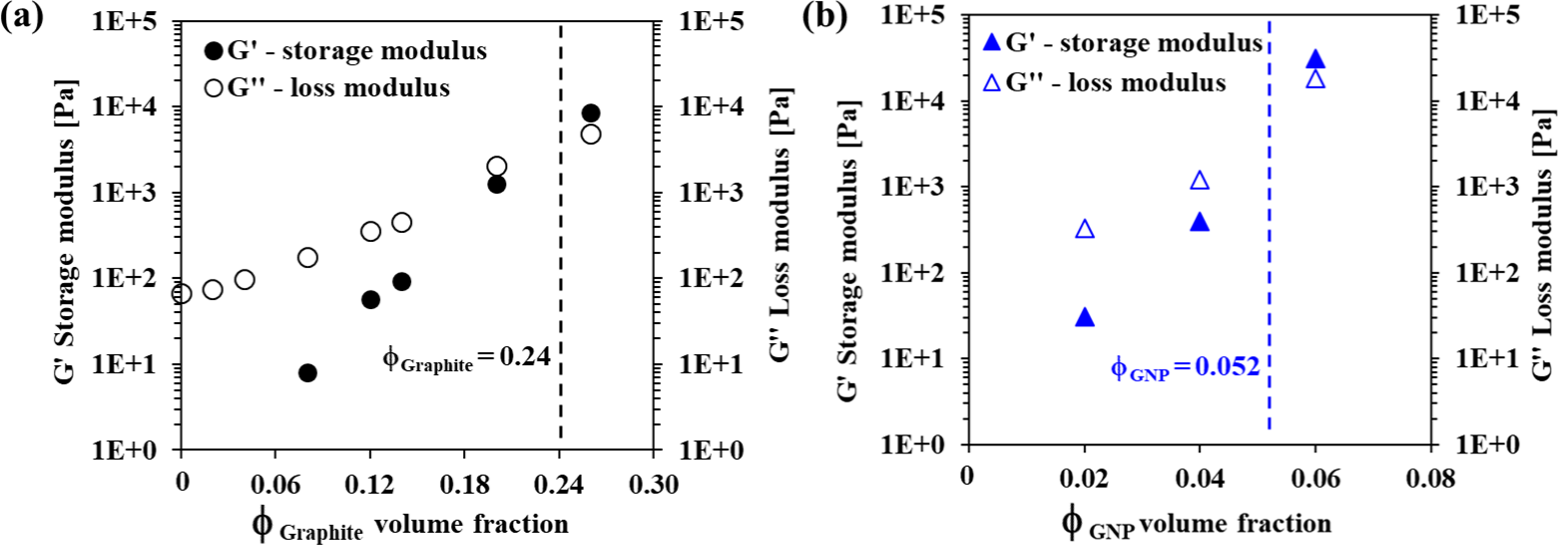
Storage modulus (G', solid symbols) and loss modulus (G', open symbols) as functions of the volume fraction of graphite (a) and GNP fillers (b) recorded at an oscillating frequency of 1 Hz and temperature of 25 °C. The dashed lines indicate the G'-G'' crossover volume fraction.

The viscosities of the GNP-loaded and graphite-loaded resins were characterized by an exponential increase above a critical filler concentration. The viscosity of the GNP–epoxy system began to increase exponentially at 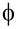
_GNP_ = 0.05, which was a much lower value than that for the graphite–epoxy system (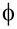
_Graphite_ = 0.24; Figure 2b). At that point, the rheological behavior of both the GNP-loaded and graphite-loaded composites changed from liquid-like to solid-like, as indicated by the dynamic modulus (see also the crossover in Figure 3, vide infra). This behavior could be fitted to the semiempirical Krieger–Dougherty (K–D) model for the relative viscosity, η_r_, in this system [47–48]:

[2] $$\eta_{\text{r}}\text{=}\frac{\eta_{\text{Composite}}}{\eta_{\text{Matrix}}}\text{=}{\left(1-\frac{\phi_{\text{Filler}}}{\varphi_{\text{M}}}\right)}^{-{\left[\eta \right]}_{\text{Filler}}\cdot \varphi_{\text{M}}}$$

where η_Composite_ and η_Matrix_ are the measured viscosities with and without the filler, respectively. The intrinsic viscosity of the filler, [η]_Filler_, represents the contribution of the filler to the composite’s viscosity, and is defined as the linear slope of the relative viscosity vs 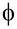
_Filler_ curve at low volume fractions, i.e., η_r_(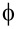
_Filler_ → 0) ≈ 1 + [η]_Filler_ · 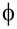
_Filler_. The critical volume fraction, φ_M_, relates to the initiation of the filler–filler interactions, where the viscosity starts increasing exponentially; this value therefore also defines the limit of the composite workability. The φ_M_ value may thus be used as a measure of the composite workability, with values higher than φ_M_ indicating that processing will be difficult. The φ_M_ of the graphite-containing composites is one order of magnitude higher than that of the GNP due to its lower aspect ratio (Figure 2b and Table 1 in the Experimental section), thereby expanding the workability range of the graphite-containing composites to higher volume fractions of the filler compared to those of GNP-loaded composites [41,48–50], in line with previously studied silicone rubber systems [41].

The viscoelasticity of a composite may be described by the dynamic moduli, G' (storage modulus) and G'' (loss modulus), which strongly depend on the volume fraction of the filler (Figure 3). A modulus ratio G''/G' > 1 reflects a more viscous material, while G''/G' < 1 indicates a more elastic material [50–51]. A crossover volume fraction (indicated by dashed lines in Figure 3) was detected for both the GNP-loaded and graphite-loaded epoxy composites at volume fractions of 0.052 and 0.24, respectively. These values agree with the critical volume fractions, φ_M_, obtained for each filler (Figure 2b), implying that the K–D model may be used as an effective prediction tool for the crossover volume fraction in this system.

In summary, the enhancement of the TC upon loading of a single filler into composites is affected by the type, dimensionality and especially the aspect ratio of the filler [52]. It has been demonstrated that loading the epoxy matrix with a high-aspect-ratio filler (e.g., GNP) results in greater TC enhancement [53] compared to loading with an isotropic filler (e.g., graphite; Figure 2a). In addition, homogenous filler dispersion in a continuous matrix results in the enhancement of the TC of the composite. However, a high-aspect-ratio filler can also reduce the TC by significantly increasing the composite viscosity (reducing workability) [52,54–55], which results in entrapping thermally insulating air bubbles within the composite [49,56], thereby increasing the contact resistance. To overcome these issues, we prepared a hybrid system consisting of both fillers, as described below.

### Thermal conductivity and rheology of hybrid composites

**Figure 4:**
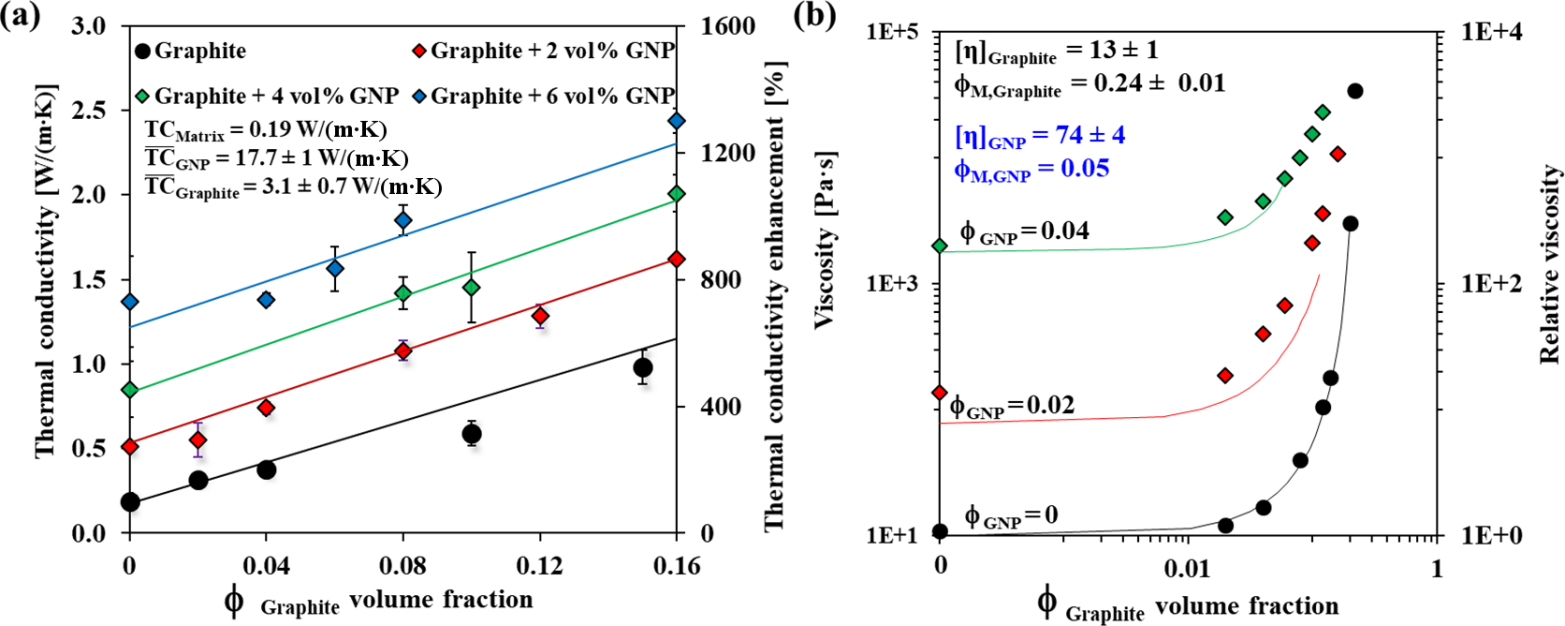
Thermal conductivity (a) and viscosity (b) of hybrid composites (at constant shear rate 0.1 [1/s], *T* = 25 °C) as a function of the graphite volume fraction. Each color represents a different constant GNP volume percent. The lines in (a) are fits to the ROM model (Equation 1), and those in (b), to the modified K–D model (Equation 2). The fitting parameters are shown in each panel.

We explored the thermal conductivity and rheology of hybrid composites in which two fillers, namely, graphite and GNP, were loaded into the epoxy matrix. We found that the hybrid filling approach makes it possible to enhance the TC value while maintaining the composite workability more effectively than with a single-filler composite. When both fillers were introduced into the epoxy resin, the changes in TC caused by varying the loading of one filler were independent of the loading of the other filler (Figure 4a), in line with the ROM model (Equation 1), which demonstrates an excellent fit to the results (Figure 4b). The TC enhancement of the hybrid filler system was far superior to that obtained with the single-filler systems (600–1200% vs ≈400%), in agreement with the literature [56–60]. Moreover, the hybrid filler system allows the composite to remain in a liquid-like phase at a higher total volume fraction of filler compared to single-filler system (22 vol % vs ≈6 vol %). The measured thermal diffusivity values of our system (Supporting Information File 1, Section S2) are in agreement with previously reported measurements of systems with similar loading [61].

The viscosity behavior of the hybrid system as a function of graphite volume fraction demonstrated a similar behavior to that shown for a single filler (Figure 4b and Figure 2b). We found that the values of φ_M_ and [η] of the two fillers obtained for the single-filler composites (Figure 2b) were also applicable to the hybrid composite, when fitting the viscosity curves (Figure 4b) to a modified version of the K–D model that takes a multiple filler system into account [62]:

[3]
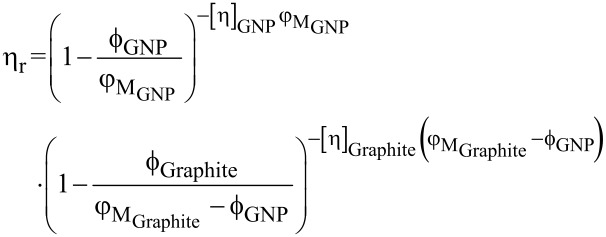


The overall relative viscosity of the hybrid includes contributions of each filler’s relative viscosities, considering that in a hybrid composite, the volume occupied by one filler is not available for the other. Equation 3 may be used for hybrid composites having two fillers substantially differing in size (by at least one order of magnitude), and is therefore applicable for our system (see Table 1 in the Experimental section). In such a case, the drag on the larger filler particles exerted by the composite medium (small filler particles and liquid) is similar to the drag they would encounter when passing through a neat liquid (without small particles) of the same density and viscosity [41,63].

The intrinsic properties of the single and hybrid systems were in good agreement for both TC and rheology: For TC, there was good agreement between the values of the calculated (Equation 1) effective conductivities for both the single (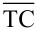
_GNP_ and 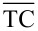
_Graphite_) and the hybrid systems (Figure 2a and Figure 4a). Similarly, the K–D parameters for both single and hybrid systems (Figure 4b) were also in good agreement, indicating that they are true values that represent the net contribution of the filler to the TC or rheology of the matrix.

**Figure 5:**
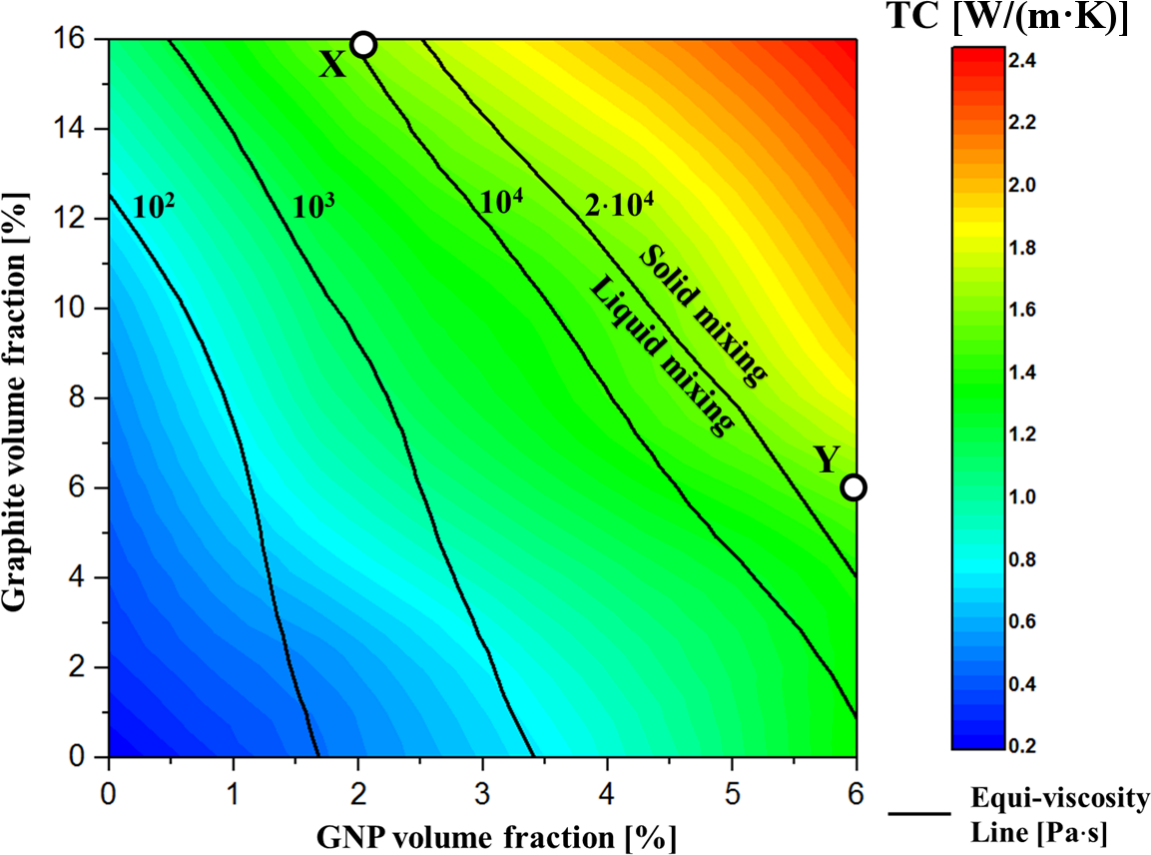
Phase diagram showing thermal conductivity (color map) and viscosity (black equi-viscosity lines) produced by interpolation of the experimental data in Figure 4a and 4b. The compositions of the samples used for functional tests (X and Y) are marked with circles.

The GNP:graphite ratio and the volume fraction of each filler contribute valuable design parameters to the thermal conductivity and the viscosity of the composite (Figure 4). Therefore, to provide a practical tool for optimizing the hybrid polymer composite composition, we constructed a phase diagram in which equi-viscosity lines are drawn over a TC vs graphite–GNP plot (Figure 5). A similar approach was previously employed for a silicone rubber system [41]. The phase diagram facilitates the design of composites containing a graphite–GNP mixture with a particular thermal conductivity, while simultaneously enabling the control of the viscosity. In addition, the phase diagram shows where the hybrid composition field is separated into solid mixing and liquid mixing, where the solid-mixing phase refers to extremely high viscosity values (not measurable with a rheometer, >20 kPa·s), and viscosities lower than this value are termed liquid-mixing. The phase diagram shows two specific composites (termed X and Y) with different hybrid compositions that yield nearly identical TC values (Figure 5). Their rheological properties are, however, quite different, with sample X lying in the liquid-mixing region and Y in the solid-mixing region, as described in Table 2. We thus show that we can produce hybrid composites having the same TC values but completely different compositions, thereby providing the capability to tune the composite workability (solid vs liquid mixing). This point is discussed further in the following section (Functional testing).

### Functional testing

The above findings indicate that for a given TC we can tune the composite workability by adjusting the hybrid composition. Indeed, we showed that completely different compositions can yield the same TC (Figure 5). To investigate this idea further, we simulated an interface in an electronic device by applying a hybrid composite as a TIM between two pieces of copper. Samples were prepared with approximately identical bulk TC values (≈1.6 W/(m∙K)) but with different viscosities (liquid mixing (sample X) and solid mixing (sample Y), see Table 2, and Figure 5) with the aim to assess the difference in applicative TC performances caused by the different rheological behaviors of the two samples.

**Figure 6:**
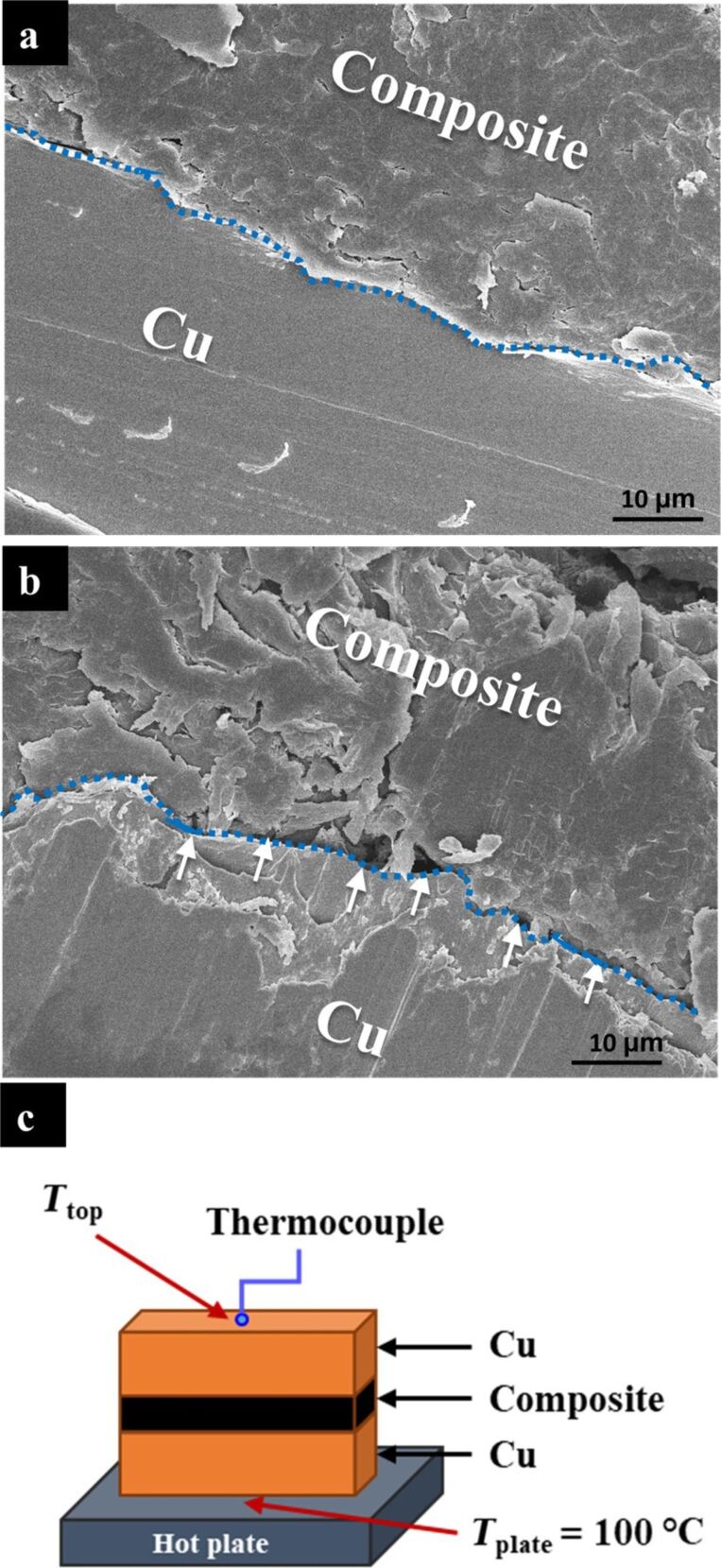
SEM micrographs of the copper–composite interfaces in (a) the lower viscosity system (sample X, 2 vol % GNP and 16 vol % graphite) and (b) the higher viscosity system (sample Y, 6 vol % GNPs and 6 vol % graphite), both with the same TC (see Figure 5). The defects at the interface are indicated with white arrows in (b). (c) Scheme of the measuring system used for the functional tests.

**Figure 7:**
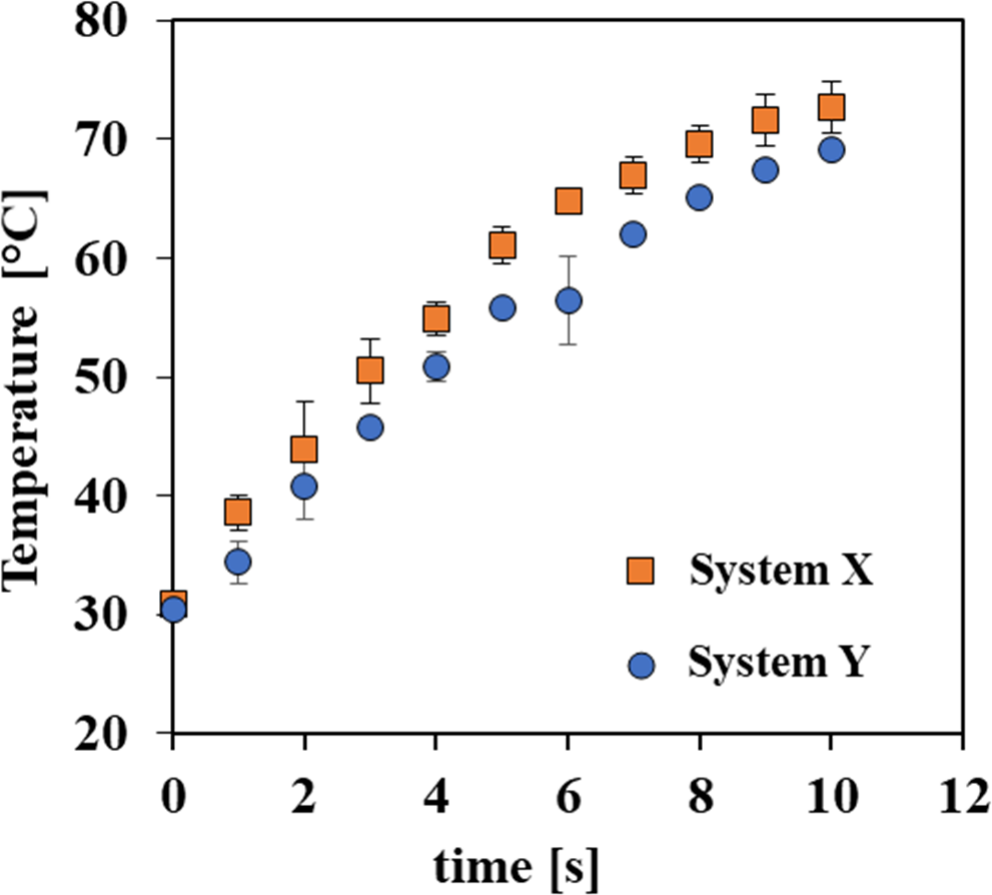
Top surface temperature (*T*_top_ in Figure 6c) of samples X and Y (Table 2) as a function of time. Measurements were recorded starting from *T*_top_ = 30 °C.

A SEM micrograph of a cross-section of sample Y (the higher viscosity TIM, solid mixing) showed interfacial defects in the form of voids between the Cu plates and the composite (Figure 6b). These voids explain the significantly increased contact resistance in this sample. These defects are not detected for the lower-viscosity sample X (Figure 6a), indicating that the higher viscosity not only impaired the composite’s workability but also reduced its overall performance as a TIM. To validate this premise, both samples were placed on a hot plate maintained at 100 °C and *T*_top_ was recorded (see Figure 6c). It was found that the temperature rise in sample X was slightly (but consistently) faster than that in sample Y (Figure 7), most probably due to the higher contact resistance in sample Y, which may be an outcome of the entrapment of air bubbles in the interface, as seen in Figure 6b.

## Conclusion

In prior research [41] we showed that loading a matrix with a hybrid graphite–GNP fillers enables one to tailor both the thermal and rheological properties of a composite material. In this work we prove that such a composite constitutes a suitable platform for thermal management applications by facilitating both fine tuning of the thermal properties and good TIM workability in a highly useful epoxy matrix.

Increased TIM viscosity resulted in increased contact resistance with the surface to which it was applied due to the formation of voids at the interface. In addition, it was found that the critical volume fractions, φ_M_, of both the GNP and the graphite fillers agree with the G'–G'' crossover volume fractions. Finally, we demonstrated that the hybrid approach, as experimentally expressed in a phase diagram (Figure 5), may be used to design a composite with a specific TC and sufficient workability, as demonstrated in a functional test of the hybrid TIM.

## Experimental

### Materials

The major properties of the fillers used in this study, namely, GNPs (grade H15-GNPs, xG-Sciences) and graphite powder (crystalline, −300 mesh, Alfa Aesar) are shown in Table 1. The epoxy matrix was comprised of diglycidyl ether of bisphenol A (EPON 828, Momentive) and a polyether triamine cross-linker (JEFFAMINE T-403, Momentive), both used as received.

**Table 1:** Relevant properties of the fillers used in this study.

System	X (Liquid mixing)	Y (Solid mixing)

composition	2 vol % GNP 16 vol % graphite	6 vol % GNP 6 vol % graphite
thermal conductivity (k)	1.62 W/(m∙K)	1.56 W/(m∙K)
viscosity at a constant shear rate of 0.1 [1/s]	10.7 kPa·s – liquid mixing	>20 kPa·s – solid mixing

**Table 2:** Composition and viscosity of two hybrid GNP–graphite composites (X and Y) having nearly identical thermal conductivities but different viscosities (see also Figure 5).

	Diameter [µm]	Aspect ratio	Thermal conductivity (*k*) [W/(m∙K)]

graphite	27 ± 4	1	100–500 [64]
graphene nanoplatelets	19 ± 3	>100	5000 [65]

### Composite preparation

The epoxy resin and hardener (10:4 epoxy:hardener; 15 g total) were loaded into a planetary centrifugal mixer (Thinky, AR-100) in a similar manner as in [41]. The filler material was added gradually (0.5 g at a time) to the mixer, which was operated at 2000 rpm (rotation + revolution) until the filler was completely incorporated. Two zirconia balls, 10 mm in diameter, were added to the mixing container to enhance the compression forces during the mixing process, resulting in the formation of homogeneous high-viscosity dispersions.

Mixing was continued for an additional 10 min after the required volume fraction had been reached, and the mixture was then deaerated (revolution, 5 min at 2000 rpm). Thereafter, the zirconia balls were removed, and the composite was cast into silicone molds (6 mm diameter and 0.6–1.8 mm thickness). The air bubbles trapped in the material were removed by vacuum treatment (10 mbar and 40 °C for 10 min), and the material was subsequently cured for 20 h at 80 °C.

### Thermal conductivity (TC)

The TC of the matrix (TC_matrix_) and of the composites (TC_composite_) was measured by differential scanning calorimetry (DSC, Mettler Toledo Star system operated under a N_2_ flow of 80 mL/min and equipped with 70 μL alumina crucibles) [24–25]. The complete measurement procedure (experimental error <5%) is detailed in the Supporting Information File 1, Section S3.

### Thermal diffusivity (TD)

The TD of the samples were measured by a thermal constants analyzer (TPS 500s, Hot Disk, Sweden) that is based on a transient plane source (TPS [66]) technique. The method requires a transiently heated plane sensor, which consists of an electrically conducting pattern in the shape of a double spiral. This spiral is sandwiched between two thin sheets of an insulating material (kapton). When performing a TD measurement, the plane Hot Disk sensor is fitted within the two composite samples. While heating up, the sensor measures the temperature increase inside the sample over time. The time-dependent change in temperature is used to calculate the TD and thermal conductivity of the measured material. The measurements were conducted in air at 25 °C [67].

### Electron microscopy and energy-dispersive X-ray spectroscopy (EDS)

Cross sections of composite samples were imaged by a high-resolution cold FEG scanning electron microscope (SEM, JSM-7400F, JEOL) equipped with an energy dispersive X-ray spectroscopy (EDS) instrument (Noran Vantage) operated in secondary electron mode at an accelerating voltage of 10 kV. EDS elemental analyses were performed on the same samples (Section S4 in Supporting Information File 1).

### Rheology

The rheological properties of the epoxy resin (prior to addition of the hardener) were determined with a rheometer (TA instruments, AR2000) operated in cone and plate arrangements (stainless steel cone, with a 40 mm diameter and a 4° cone angle) at 25 °C. The shear rate was swept between 0.01–100 1/s, and each measurement was performed at steady-state flow at a shear rate of 0.01 1/s to extract the viscosity [51,68].

### Functional thermal conductivity testing

A liquid layer of uncured composite was sandwiched between two 200 µm thick copper sheets, and pressed to a thickness of 540 ± 15 µm using a table clamp. The sample was then hardened at 80 °C for 24 h. The sandwich was cooled to room temperature and then placed on a hot plate, kept at 100 °C; a thermocouple was attached to the upper copper surface, and the temperature was recorded at intervals of 1 s.

## Supporting Information

File 1 Thermal conductivity measurement and elemental analysis.
