# Supplementary material for: Graphene–graphite hybrid epoxy composites with controllable workability for thermal management
Source: Beilstein J Nanotechnol. 2019 Jan 8;10:95–104. doi: 10.3762/bjnano.10.9 (PMC6334801; doi:10.3762/bjnano.10.9)
Supplement: File 1 — Thermal conductivity measurement and elemental analysis. [file Beilstein_J_Nanotechnol-10-95-s001.pdf]

BEILSTEIN JOURNAL OF NANOTECHNOLOGY

Supporting Information

for

Graphene–graphite hybrid epoxy composites with controllable workability for thermal management

Idan Levy, Eyal Merary Wormser, Maxim Varenik, Matat Buzaglo, Roey Nadiv and Oren Regev

Beilstein J. Nanotechnol. **2019,** *10,* 95–104. doi:10.3762/bjnano.10.9

Thermal conductivity measurement and elemental analysis

License and Terms: This is a supporting information file under the terms of the Creative Commons Attribution License (http://creativecommons.org/licenses/by/4.0). Please note that the reuse, redistribution and reproduction in particular requires that the authors and source are credited.
The license is subject to the *Beilstein Journal of Nanotechnology* terms and conditions: (https://www.beilstein-journals.org/bjnano)

S1. Characterization of the fillers are imbedded in the epoxy matrix

The mean lateral dimension, $\bar{L}$,of the filler particles was analyzed from SEM images (e.g., Figure 1). The mean lateral dimension is defined by the square root of the graphene area: $\bar{L} = \sqrt{\min \cdot \max lenghts}$ as shown in Figure S3. A total of 100 particles were measured for each filler.

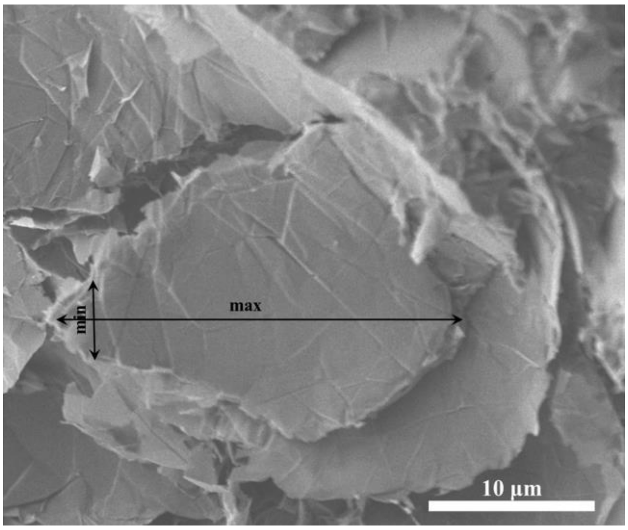

Figure S1: SEM image of a single GNP filler shown is an analysis of an $\bar{L}$ average length (mean lateral dimension).

The sizes of the different fillers, when imbedded in the epoxy matrix, are 19 ± 3 μm and 27 ± 4 μm for the GNP and the graphite, with the size distribution presented in Figure S4a-b.

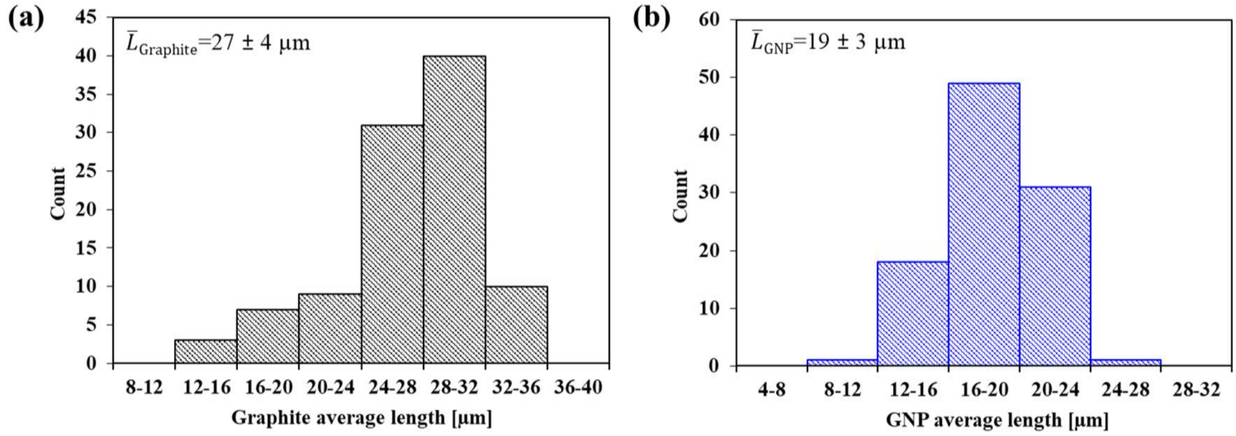

Figure S2: Size distribution of (a) graphite and (b) GNP fillers (N_i = 100 particles for each filler).

S2. Thermal diffusivity values

Thermal diffusivity (TD) was measured for selected samples, as shown in Table S1. The **TC values** were measured using both DSC (described in Section S3, below) and the Hot-Disk techniques (experimental section, main text) and provide similar results. Table S1 shows that TD values are proportional to TC values, i.e., an increase in TC values leads to an increase in TD values, which is consistent with the specific heat results that are almost unaffected by the composition of the composite material. In addition, the values shown in the table below correspond to those reported in the literature [1].

Table S1: Thermal properties of single and hybrid GNP-graphite composites.

Composition	TC (DSC) [W/(m·K)]	TC (Hot-disk) [W/(m·K)]	Thermal diffusivity (Hot-disk) [mm^2/s]	Specific heat [MJ/(m^3·K)]
**2 vol % GNP + 4 vol % graphite**	0.74 ± 0.04	0.79 ± 0.01	0.53 ± 0.02	1.50 ± 0.03
**4 vol % GNP**	0.85 ± 0.17	1.03 ± 0.02	0.72 ± 0.02	1.43 ± 0.03
**10 vol % graphite**	0.59 ± 0.07	0.56 ± 0.01	0.37 ± 0.01	1.53 ± 0.02
**2 vol % GNP**	0.51 ± 0.03	0.63 ± 0.01	0.41 ± 0.01	1.54± 0.01

S3. Thermal Conductivity (TC) measuremnets by DSC

TC measurement procedure was adopted from our previous work [2]. Liquid gallium (150 mg) was poured into a 70 μL alumina crucible, which was placed on top of a disc-shaped sample. To prevent differences in interface resistance between the experiments [3], the spaces between the crucible-sample and sample-sensor interfaces were covered with a thin layer (<0.5 μL) of silicone heat-transfer oil (CLEARCO, DPDM-400).

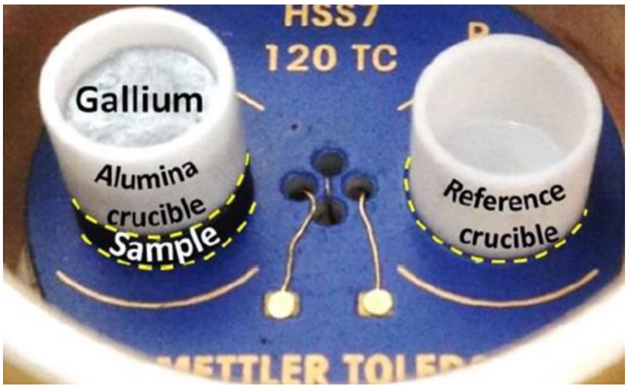

Figure S3: Sample arrangement on the DSC sensor. The empty reference crucible was placed on the right. An identical crucible containing gallium was placed on top of the sample on the left. The yellow dashed lines indicate the silicone heat-transfer oil. Adapted with permission from [2], copyright 2017 American Chemical Society.

This sample was heated from 28 °C to 37 °C at a heating rate of 0.5 °C min^{-1}. When gallium reaches its melting temperature, 29.76 °C, the temperature at the top of the sample disc remains constant, until the gallium is fully melted. The temperature at the lower surface of the sample and the heat flowing into it are measured by the DSC. The TC of the sample is calculated from the temperature differential between the upper and lower surfaces (ΔT) of the disc and the heat flow (Q):

$$\frac{\Delta T}{Q} = R_{\mathrm{T}} + \frac{1}{TC} \cdot \frac{h}{A} \quad \text{(S1)}$$

where R_{T} is the combined thermal resistance at the sensor/sample and sample/crucible interfaces; h is the thickness of the sample disc; and A is the cross-sectional area of the sample.

After measuring at least three randomly orientated samples with different thicknesses, TC and R_T were determined by linear regression (Figure S2).

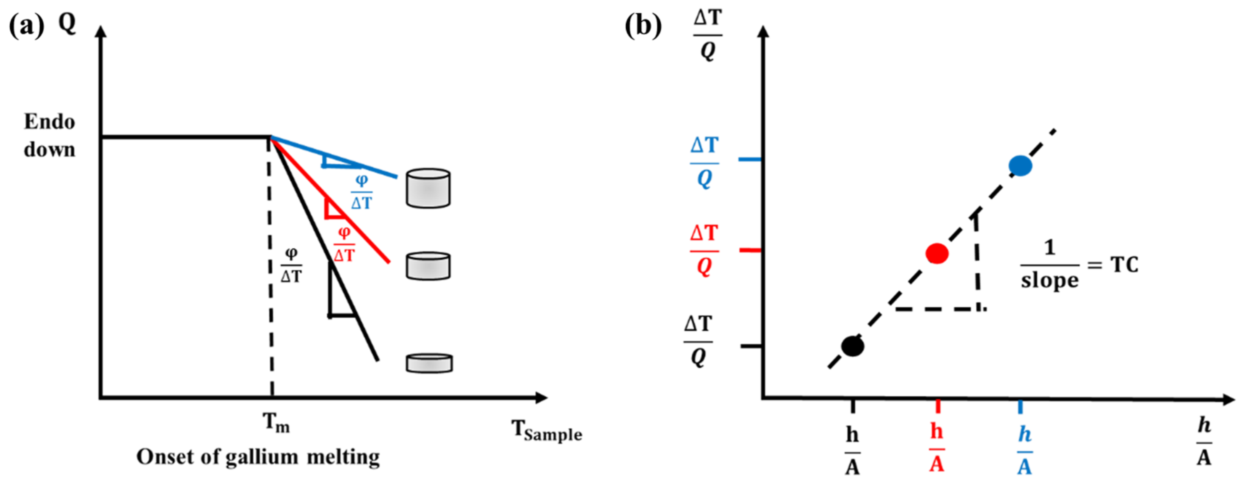

Figure S4: Schematic representation of TC measurement. At least three samples with varying thicknesses were measured, and the slope of the melting curve was determined ($\frac{Q}{\Delta T}$) for each one (a). Thicker samples showed higher thermal resistance and, thus, their temperatures changed more gradually (lower $\frac{Q}{\Delta T}$). The slope found by plotting the inverse of the melting-curve slope ($\frac{\Delta T}{Q}$) versus the thickness to cross-section ratio ($\frac{h}{A}$) yielded the sample's inverse TC (b). Adapted with permission from [2], copyright 2017 American Chemical Society.

S4. Elemental Analysis of SEM micrographs

Elemental analysis of the SEM micrographs was carried out using EDS for the samples represented in the micrographs (Figure 5a and 5b, in the main text) to distinguish between the composite and the Cu plates.

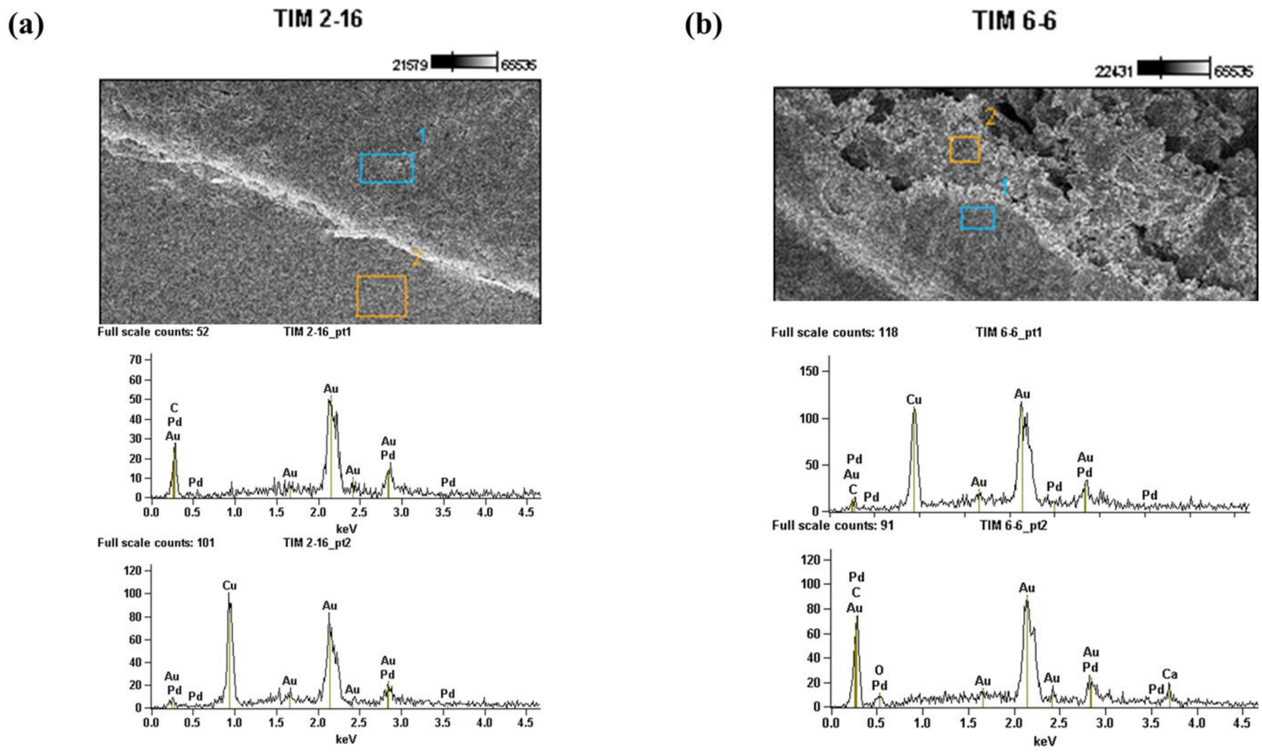

Figure S5: (a) EDS of a composite containing 2 vol % GNP and 16 vol % graphite, at a magnification of ×250; (b) EDS of a composite containing 6 vol % of both GNP and graphite, taken at ×450.

References

1. Chiguma, J.; Johnson, E.; Shah, P.; Gornopolskaya, N.; Jones Jr, W. E., *Open J. Compos. Mater.* **2013,** *3*, 51. doi:10.4236/ojcm.2013.33007
2. Varenik, M.; Nadiv, R.; Levy, I.; Vasilyev, G.; Regev, O., *ACS Appl. Mater. Interfaces* **2017,** *9*, 7556-7564. doi:10.1021/acsami.6b14568
3. Hakvoort, G.; Van Reijen, L.; Aartsen, A., *Thermochim. Acta* **1985,** *93*, 317-320. doi:10.1016/0040-6031(85)85081-4
